# Cancer Risk of Anti-TNF-α at Recommended Doses in Adult Rheumatoid Arthritis: A Meta-Analysis with Intention to Treat and per Protocol Analyses

**DOI:** 10.1371/journal.pone.0048991

**Published:** 2012-11-14

**Authors:** Guillaume Moulis, Agnès Sommet, Johana Béné, François Montastruc, Laurent Sailler, Jean-Louis Montastruc, Maryse Lapeyre-Mestre

**Affiliations:** 1 CHU Toulouse, Service de Médecine Interne, Toulouse, France; 2 CHU Toulouse, Service de Pharmacologie Clinique, Centre Midi-Pyrénées de PharmacoVigilance, de Pharmacoépidémiologie et d’Informations sur le Médicament, Toulouse, France; 3 Inserm, UMR1027, Toulouse, France; 4 Université de Toulouse III, UMR1027, Toulouse, France; 5 CHU Lille, Centre Nord-Pas de Calais de PharmacoVigilance, Lille, France; 6 Université de Lille II, Lille, France; University of Hong Kong, China

## Abstract

**Background:**

The risk of malignancies on TNF-α antagonists is controversial. The aim of this survey was to assess cancer risk on TNF-α antagonists in adult rheumatoid arthritis patients, including the five marketed drugs (infliximab, etanercept, adalimumab, golimumab and certolizumab) used in line with the New Drug Application. Furthermore, the relative interest of modified intention to treat or per protocol analyses to assess such sparse events remains unknown.

**Methodology/Principal Findings:**

Data sources were MEDLINE, CENTRAL, ISI Web of Science, ACR and EULAR meeting abstracts, scientific evaluation of the drugs leading to their marketing approval, and clinicaltrials.gov, until 31 December 2012.We selected double-blind randomized controlled trials in adult rheumatoid arthritis patients, including at least one treatment arm in line with New Drug Application. We performed random effect meta-analysis, with modified intention to treat and per protocol analyses. Thirty-three trials were included. There was no excess risk of malignancies on anti-TNF-α administered in line with New Drug Application in the per protocol model (OR, 0.93 95%CI[0.59–1.44]), as well as in the modified intention to treat model (OR, 1.27 95%CI[0.82–1.98]). There was a non-significant tendency for an excess non-melanoma skin cancer risk in both models (respectively, 1.37 [0.71–2.66] and 1.90 [0.98–3.67]). With fixed effect Peto model restricting to trials during at least 52 weeks, the overall cancer risk was respectively 1.60 [0.97–2.64] and 1.22 [0.72–2.08]. Whatever the model, modified intention to treat analysis led to higher estimations than per protocol analysis. The later may underestimate the treatment effect when assessing very sparse events and when many patients dropped out in placebo arms. In metaregression, there was no differential risk among the five drugs.

**Conclusions/Significance:**

This study did not find any evidence for an excess cancer risk on TNF-α antagonists in adult rheumatoid arthritis patients, but an excess cancer risk after several years of exposure cannot be ruled out. Both modified intention to treat and per protocol analyses should be presented in such safety analyses.

## Introduction

The risk of malignancies on anti-TNF-α therapies is controversial, since TNF-α exerts both pro and anticancer properties [1]. Meta-analyses (MAs) of randomized controlled trials (RCTs) have led to conflicting results. These discrepancies may be due to methodological differences. Indeed, the MAs which have included the greatest number of trials evaluated anti-TNF-α drugs regardless of their indication, while baseline risk depending on the disease was not comparable [2], [3]. Despite adjustment on the condition, some heterogeneity remains and it is difficult to conclude on the cancer risk regarding a specific indication for which TNF-α antagonists are widely used, such as rheumatoid arthritis. Nevertheless, five MAs were restricted to adult rheumatoid arthritis patients [4]–[8]. Mean number of RCTs included in these MAs was 10.6. Indeed, few MAs used an extended search for unpublished RCTs [4], [6]. Furthermore, some of these studies included open-label extension periods of RCTs, resulting in a possible diagnosis bias. Indeed, in the absence of double blinding, patients on anti-TNF-α drugs might be more accurately screened for malignancies than others. Moreover, these studies are far removed from usual standard care: all but two MA pooled data from patients exposed to anti-TNF-α regardless to the prescribed dose [4], [7] and some MAs included RCTs using unusual anti-TNF-α administration, *e.g.* intra-articular [3]. Eventually, only one MA included the five marketed TNF-α antagonists, and it was whatever the underlying disease [3].

**Figure 1:**
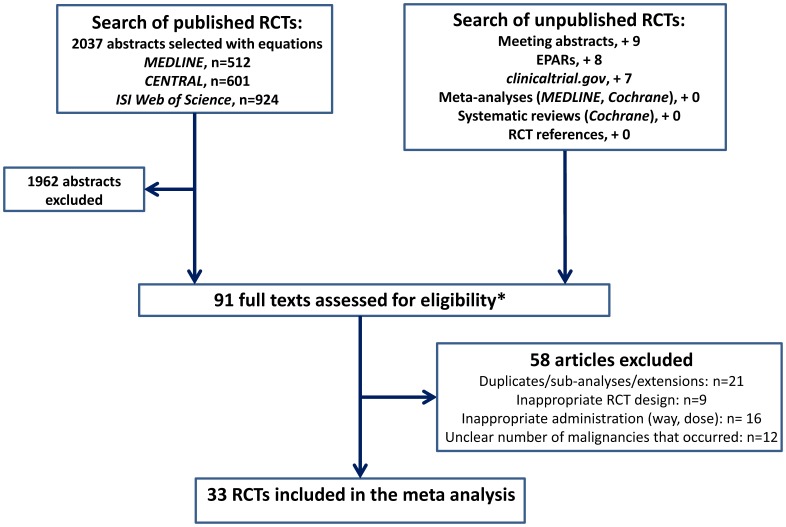
Flowchart illustrating the trials selection. *8 duplicates in the search of unpublished RCTs.

So, we conducted a new MA of RCTs to assess the cancer risk of TNF-α antagonists in adult rheumatoid arthritis patients, including the five drugs marketed. Our work was strictly restricted i) to the arms in accordance with New Drug Application (NDA), ii) to the double-blind period, to avoid diagnosis bias, and iii) to anti-TNF-α naive patients, to accurately measure the exposure. The main objective was the assessment of the overall cancer risk. Secondary objectives focused on the risk assessment of solid cancers (including and excluding skin cancers), haematological neoplasms, cutaneous cancers overall, non-melanoma skin cancers (NMSCs) and melanomas. We also performed a separate MA restricted to doses lower and higher than those of NDA to investigate a possible dose-effect relation. Lastly, we assessed the putative different risk among the five marketed TNF-α antagonists.

Previously published MAs assessing cancer risk on anti-TNF-α have been conducted in intention to treat analysis or modified intention to treat analysis (mITT). However, safety surveys can be conducted in per protocol (PP) analyses so as to give a maximal estimation of the risk and to make sure that all included patients have been exposed to the drug during all the time of the survey. This PP analysis should be justified in a safety analysis thus we are interested in patients truly exposed to the drug. Nonetheless, PP and ITT MAs could result in very conflicting results. The direction and extent of these discrepancies are unpredictable [9]. In this MA, we compared the results of mITT and PP analyses.

## Methods

We conducted MA on pooled data. The methodology employed was in line with PRISMA guidelines [10].

### Search Strategy

The search of published RCTs until 31 December 2010 was conducted in MEDLINE, CENTRAL and ISI Web of Science without limit of language (see equations in Method S1). Two independent evaluators (GM and FM) performed a first selection of RCTs reading the abstracts. Discrepancies between the two evaluators were solved by consensus. A third evaluator (AS) intervened in case of persistent disagreement.

The search of unpublished trials was assessed by reading i) the references of published RCTs selected at the first step described above, ii) the references of systematic reviews and MAs assessing efficacy or safety of TNF-α antagonists in rheumatoid arthritis published in MEDLINE and in the Cochrane database, iii) the American College of Rheumatology meeting abstracts (1990–2010) and the European League Against Rheumatism meeting abstracts (2001–2010), iv) the scientific evaluation of the drugs leading to European marketing approval and v) request on clinicaltrials.gov.

**Figure 2:**
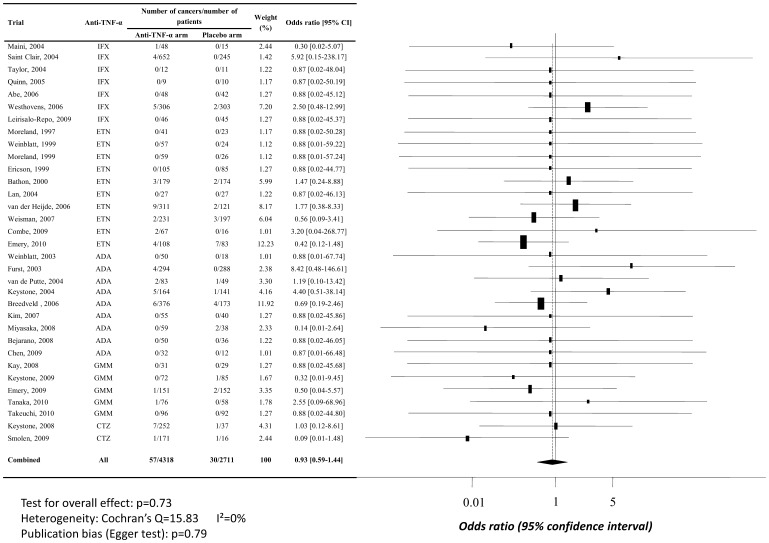
Random per protocol meta-analysis of overall cancer risk when TNF-α antagonists are used at doses in line with New Drug Approval. Abbreviations: ADA, adalimumab; CTZ, certolizumab pegol; ETN, etanercept; GMM, golimumab; IFX, infliximab; 95% CI, 95% confidence interval.

RCTs selected after this first step were independently evaluated by two evaluators (GM and JB) thanks to an *a priori* established evaluation grid recapitulating inclusion and exclusion criteria. Discrepancies were also solved by consensus. A third evaluator (AS) intervened in case of persistent disagreement. The RCTs excluded at this step were described with reasons for exclusion.

### Inclusion Criteria

Patients were anti-TNF-α naive adults suffering from rheumatoid arthritis defined in most RCTs with the 1987 modified ACR criteria [11]. RCTs were placebo-controlled, double blind, and evaluated one of the five anti-TNF-α drugs (infliximab, etanercept, adalimumab, golimumab, and certolizumab pegol) either alone or associated with methotrexate. The dose, frequency and way of administration should be in accordance with NDA. When patients had been previously exposed to anti-TNF-α before the beginning of a trial, they were excluded. The methodological quality of RCTs was assessed thanks to the Delphi list and the Oxford quality scale; a score <3 in the latest leaded to exclusion of the study [12], [13]. The primary outcome was the overall number of cancers that occurred. The secondary outcomes were the numbers of solid cancers (including and excluding skin cancers), haematological neoplasms, cutaneous cancers overall, NMSCs, and melanomas. The number of cancers should be indicated at the end of the RCT or at the end of the blinding period in case of open-label extension. When patients had been exposed to a previous anti-TNF-α (patients that we excluded), we checked whether they acquired cancer during the trial (cancers that we also excluded for the analyses). In case of missing or unclear data, authors or pharmaceutical companies were systematically contacted by email or phone to provide supplementary information. In case of non-response, a second contact was attempted. Eventually, a third email was sent to both co-authors and pharmaceutical companies.

**Figure 3:**
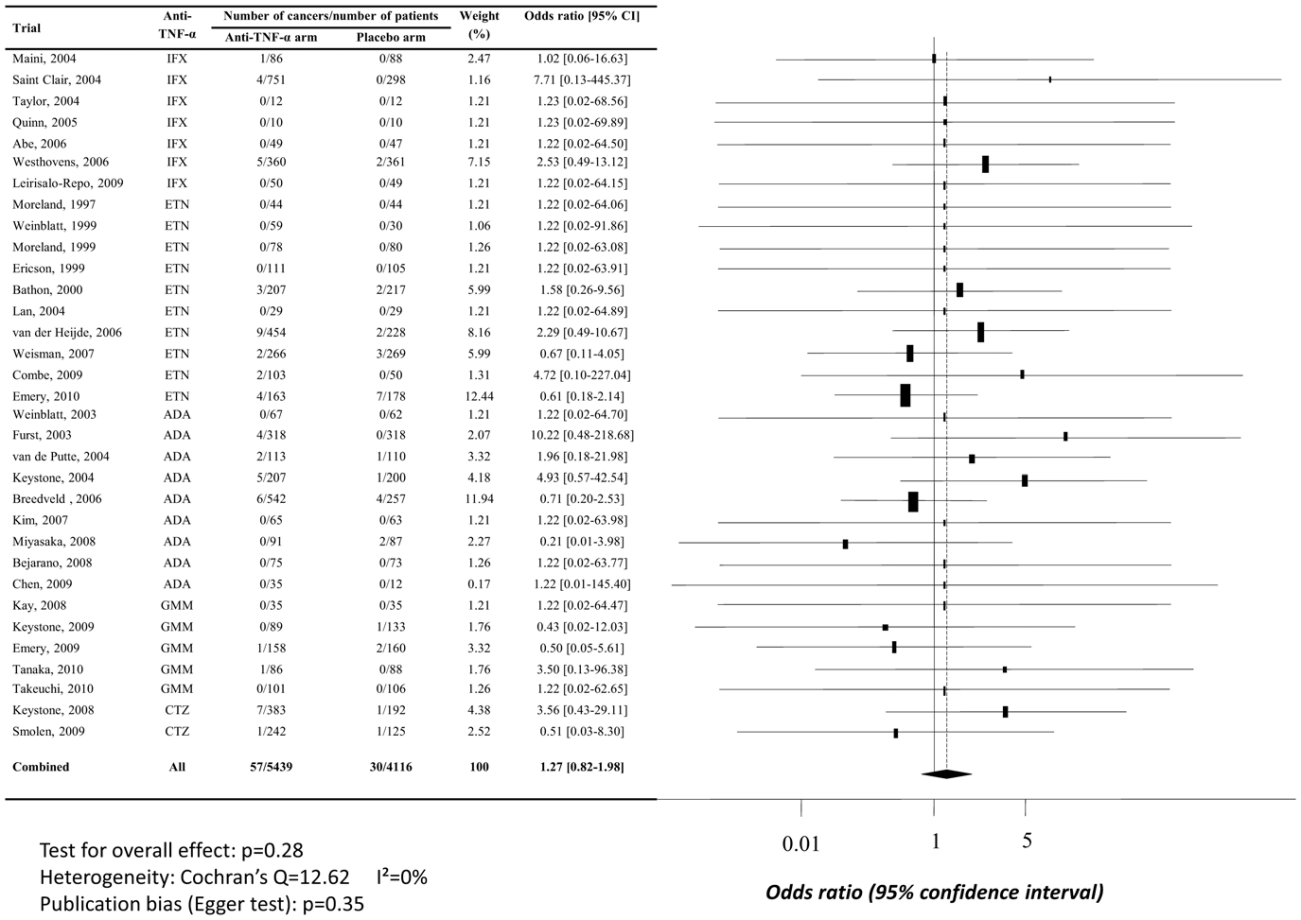
Random modified intention to treat meta-analysis of overall cancer risk when TNF-α antagonists are used at doses in line with New Drug Approval. Abbreviations: ADA, adalimumab; CTZ, certolizumab pegol; ETN, etanercept; GMM, golimumab; IFX, infliximab; 95% CI, 95% confidence interval.

### Statistical Analyses

Random-effect MA (DerSimonian and Laird model) was conducted on pooled data. Treatment effect was estimated by odds ratios (OR) with their 95% confidence interval (95%CI). Null values were treated with empirical (“pseudo-bayesian”) continuity correction [14]. In sensitivity analysis, we tested other methods of continuity correction preferentially used in previously published MAs (adding the value 0.5, adding the value 0.01, and adding the inverse of the size of the corresponding treatment arm) [14]. We also tested fixed effect models (Mantel-Haenszel and Peto) in sensitivity analysis. Heterogeneity was assessed with Cochran’s test and I^2^ index [15]. Both PP and mITT analyses were performed. Patients who received at least one dose of treatment in each arm defined the mITT patients. The number of patients in the PP model was calculated as follows: the number of patients who completed the study with their initial treatment (patients rescued with increased doses or treatment switch were excluded), added to the number of patients who dropped out for malignancies. Sensitivity analyses were performed pooling all anti-TNF-α arms in each RCT (that is to assess cancer risk whatever the dose administered, as it has been performed in previously published MAs) and excluding the RCTs during less than 52 weeks. Indeed, the threshold of 12 weeks to develop a cancer, used in previous meta-analyses, is questionable. To look for a possible dose-effect relationship, we performed a separate MA restricted to doses lower or higher than those of NDA. Publication bias was evaluated by Egger’s test and its graphical illustration [16]. We used metaregression to assess the putative different risk of overall cancer risk among the five marketed TNF-α antagonists. The Stata 11.2™ software was used to perform analyses (StataCorp LP, College Station, TX, USA).

**Table 1:** Risk of cancers assessed by the modified intention to treat model and by the per protocol model.

Doses	Overall cancers (OR [95%CI])	Solid cancers ‡(OR [95%CI])	Solid cancers,except cutaneous‡(OR [95%CI])	Haematologicalcancers ‡(OR [95%CI])	Cutaneous cancers ‡ (OR[95%CI])	NMSC (OR[95%CI])§
**Doses in line with the NDA**						
mITT	1.27 [0.82–1.98]	1.25 [0.79–1.98]	1.34 [0.78–2.31]	0.70 [0.36–1.36]¶	1.81 [0.97–3.36]	1.90 [0.98–3.67]
Per protocol	0.93 [0.59–1.44]	0.90 [0.57–1.42]	0.97 [0.56–1.67]	0.62 [0.31–1.24]¶	1.26 [0.68–2.35]	1.37 [0.71–2.66]
**Doses superior to those of the NDA** *						
mITT	1.56 [0.78–3.12]	1.62 [0.78–3.35]	1.04 [0.43–2.48]	-	1.37 [0.55–3.38]	1.27 [0.52–3.11]
Per protocol	0.88 [0.44–1.76]	0.90 [0.43–1.87]	0.55 [0.23–1.32]	-	0.74 [0.30–1.84]	0.70 [0.28–1.72]
**Doses inferior to those of the NDA** †						
mITT	0.90 [0.28–2.88]	1.12 [0.31–3.96]	1.13 [0.30–4.29]	-	1.04 [0.22–4.82]	1.04 [0.22–4.82]
Per protocol	0.75 [0.23–2.44]	0.98 [0.27–3.55]	1.11 [0.28–4.43]	-	0.70 [0.15–3.29]	0.70 [0.15–3.29]
**All doses**						
mITT	1.24 [0.81–1.90]	1.26 [0.81–1.96]	1.03 [0.61–1.76]	0.77 [0.40–1.48]¶	1.53 [0.83–2.79]	1.44 [0.77–2.71]
Per protocol	0.89 [0.58–1.35]	0.88 [0.57–1.37]	0.69 [0.41–1.18]	0.68 [0.34–1.36]¶	1.03 [0.57–1.88]	0.93 [0.50–1.74]

Abbreviations: mITT, modified intention to treat; NDA, New drug Approval; NMSC, non-melanoma skin cancers; OR, odds ratio; 95% CI, 95% confidence interval;

* 14 trials included for overall cancer analysis.

† 7 trials included for overall cancer analysis.

‡ The trial by Miyasaka *et al*. was excluded from these analyses for the two cancers that occurred in the placebo arm were not otherwise specified.

§ These analyses excluded two trials: the one by Miyasaka *et al*. (because the two cancers that occurred in the placebo arm were not otherwise specified) and the one by Weisman *et al.* (because the three skin cancers that occurred were not otherwise specified).

¶ Publication bias.

## Results

The process of selection is illustrated in Figure 1. Seventy-five RCTs were selected among 2037 abstracts selected in the three databases. Searches in ACR and EULAR abstracts, clinicaltrial.gov and scientific discussions leading to marketing approval allowed retrieving 16 supplementary RCTs to read in detail. Reading the references of previous systematic reviews and MAs (MEDLINE, Cochrane) did not result in adding unknown RCTs.

**Table 2:** Overall cancer risk at doses in line with NDA, restricting the analysis to trials during at least 52 weeks (n = 13, continuity correction used: 0.01, I^2^ = 0).

Model	Per protocol analysis *Odds ratio [95% confidence interval]*	Modified intention-to-treat analysis *Odds ratio [95% confidence interval]*
**Random effect (DerSimonian and Laird)**	1.04 [0.58–1.87]	1.39 [0.77–2.49]
**Fixed effect (Mantel-Haenszel)** *	1.22 [0.71–2.11]	1.65 [0.96–2.85]
**Fixed effect (Peto)** *	1.22 [0.72–2.08]	1.60 [0.97–2.64]

* Restricted to trials with at least one event, n = 10.

After detailed reading with the evaluation grid, 33 RCTs were included. Excluded RCTs at this step and reasons for rejection are shown in Table S1. Twelve trials were excluded because of a lack of precision regarding the number of malignancies that occurred. Among these 12 RCTs, the authors explicitly did not wish to send us data since they were preparing a publication in three cases. In seven other cases, the trials were former RCTs identified in European Public Assessment reports or clinicaltrials.gov, for which pharmaceutical companies were unable to provide further data. Since we got very few data, they were not included in the study. Nevertheless, they were early phase trials, and as a result they might not have been included.

Included RCTs are listed and their characteristics described in Table S2. The drugs were infliximab in 7 RCTs, etanercept in 10, adalimumab in 9, golimumab in 5 and certolizumab pegol in 2. Pooling all the treatment arms (*i.e.* whatever the dose), 94 cancers occurred on TNF-α antagonist and 30 on placebo. Among the 94 cancers that occurred on TNF-α antagonist, 57 occurred at doses in line with NDA. The details of cancers that occurred in each RCT are reported in Table S3.

In the PP model, there was no excess risk of malignancies for anti-TNF-α administered in line with NDA (OR, 0.93, 95% CI [0.59–1.44]), as shown in Figure 2. In the mITT model, there was no excess risk of malignancies either for anti-TNF-α administered in line with NDA, but estimation of treatment effect was higher than in the PP model (OR, 1.27, 95% CI [0.82–1.98]), *cf.* Figure 3. Nevertheless, these differences between mITT and PP analyses was non-significant, thus 95% confidence intervals odd ratios overlap. Egger’s test did not sustained a publication bias (respectively, p = 0.79 and p = 0.35) and no heterogeneity was found (I^2^ = 0).

Results regarding the various types of cancers in both mITT and PP models are presented in Table 1. The maximal risk was for NMSCs, albeit also non-significant (OR, 1.37 95%CI[0.71–2.66] with PP estimation and OR, 1.90 95%CI[0.98–3.67] with mITT model, when TNF antagonists were used in line with NDA). We could not calculate the empirical continuity correction because of too few events regarding melanomas in all dose analyses and haematological malignancies for the doses not in accordance with NDA.

In metaregression, there were no differences among the five anti-TNF-α drugs administered in line with NDA regarding the overall cancer risk (PP model, p = 0.42, mITT model, p = 0.26).

The use of 0.5, 0.01 or “arm size weighted” continuity correction led to slightly lower treatment effect (data not shown).

Fixed effect models, restricted to trials whit at least one event (n = 18), lead to higher estimations albeit also non-significant. With Mantel-Haenszel model, the overall cancer risk at doses in line with NDA was 1.46, 95%CI[0.93–2.31] in mITT analysis and 1.09, 95%CI[0.69–1.71] in per protocol analysis (with 0.01 continuity correction, I^2^ = 0). With Peto model, odds ratio was 1.44, 95%CI[0.93–2.22] in mITT model and 1.09, 95%CI [0.69–1.72] in per protocol one (with 0.01 continuity correction, I^2^ = 0).

When restricting to RCTs during at least 52 weeks (n = 13), odds ratio for overall cancer risk at doses in line with NDA were notably higher in both mITT and in PP analyses, albeit also not significantly (Table 2). In mITT random effect analysis, the risk was 1.39 95%CI[0.77–2.49], while it was 1.60 95%CI[0.97–2.64] in fixed effect model.

## Discussion

Whatever the model used (PP or mITT), this MA of RCTs did not find an excess cancer risk on the five TNF-α antagonists used in line with NDA compared with placebo in adult rheumatoid arthritis patients.

These results are inconsistent with the first MA assessing the risk of cancer on anti-TNF-α drugs. Indeed, Bongartz *et al.* found an excess cancer risk on anti-TNF-α therapy (OR, 3.29, 95% CI [1.13–9.08]), but they only investigated infliximab and adalimumab, and included fewer RCTs including these two drugs in comparison with our present work. Moreover, they worked on data provided by pharmaceutical companies and included some cancers that occurred after the double-blind period [4]. We cannot exclude a diagnosis bias: in the absence of blinding, patients on TNF antagonists may have been screened more accurately for cancers than other patients. Askling *et al*. found an increased risk of NMSCs on anti-TNF-α [2]. This is consistent with the tendency we found at recommended doses, which was non-significant probably because of lack of power. Very recently, a MA of 29 observational studies has been conducted (among the 29, 25 were limited to rheumatoid arthritis patients) [17]. The overall cancer risk was estimated to be 0.95, 95% CI [0.85–1.05]. The authors also found a significant excess of risk only for NMSCs (four surveys, OR, 1.45, 95% CI [1.15–1.76]).

We found a pattern toward dose-effect relation regarding the overall risk of cancer (particularly in mITT analysis), albeit non-significant. This result was suspected by the first MA conducted by Bongartz *et al.*, but not by Leombruno *et al.* [4], [7]. Our results did not show such a relation when considering each type of cancer, perhaps because of lack of power.

Estimations in fixed effect models (Mantel-Haenszel and Peto) were higher than with random effect (DerSimonian and Laird) model because of exclusion of RCTs with no event. Indeed, the presence of such RCTs in random effect model lead to a tendency toward the null. Nevertheless, these RCTs with no events are important information and that’s why we kept them in first analysis with random effect model.

Albeit non-significant, mITT estimations were always superior to PP ones. Several explanations can be discussed to explain discrepancies between PP and ITT analysis. First, mITT analysis may result in a gain of power. Nevertheless, the fact that estimates of treatment effect do not vary widely when pooling all doses does not sustain this conclusion. Secondly, and more certainly, there may be a diagnosis bias in mITT analysis which may overestimate the treatment effect. Indeed, drop-out patients (particularly those lost to follow-up) cannot be diagnosed for a cancer after withdrawal from the RCT. As the number of drops out is much higher in placebo arms than in anti-TNF-α arms (loss to follow-up or dropping out for inefficacy), this would result in overestimation of the cancer risk on anti-TNF-α therapy. On the contrary, PP analysis lead to a lack of power particularly in placebo arms, but its main advantage is that it provides the certainty that all the patients included in the analysis have been exposed to placebo or anti-TNF-α during the whole trial.

PP analysis may underestimate the treatment effect in assessing very few events and in case of many drop-out patients in placebo arms. Indeed, that leads to overestimate the odds in placebo arms. For example, in a RCT reported by Keystone *et al.*, the numbers of anti-TNF-α naïve patients in the anti-TNF-α arm and in the placebo arm were respectively 383 and 192 in mITT analysis, and 252 and 37 in PP analysis [18]. Four cancers occurred in the anti-TNF-α arm, and 1 in the placebo arm. The odds in mITT analysis were 0.0104 in the anti-TNF-α arm and 0.0052 in the placebo arm and so the OR was 2. In PP analysis, the odds were 0.0158 in the anti-TNF-α arm and 0.0270 in the placebo arm and so the OR was 0.85. Furthermore, drop-out patients in placebo arms mainly withdrew from the RCT because of lack of efficacy. These patients could suffer from a more inflammatory disease, and may have had an increased baseline risk for cancer. These patients were not taken into account in PP analysis.

We assessed the risk of the five marketed TNF-α antagonists. Unlike the previously published MAs, we focused on RCTs in which the anti-TNF-α were used in line with the NDA, approaching the “real life” conditions. Methodologically, the first strength of the present study is the search of unpublished RCTs thanks to several additional sources. Second, the systematic assessment of quality and the restriction to blind period led to exclude unblinded extensions, and allowed to minimize a diagnosis bias. Third, we systematically contacted authors or sponsors in case of unclear or missing data, as previously done by Bongartz *et al*. in their MAs [4], [6]. As a result, the pitfalls of such a safety MA could be avoided [19]. Fourth, we compared mITT and PP models. Fifth, its power (33 RCTs) is widely superior to previous MAs. Sixth, our study is academic and none of the authors has any conflict of interest to declare with pharmaceutical companies marketing anti-TNF-α drugs.

Some limitations of our study should be underlined. First, we worked on pooled data, which is less accurate than individual ones. Second, the search on MEDLINE used MeSH terms and therefore may be too restrictive. Nevertheless, the other published and non-published searches allow us to think that we eventually did not miss RCTs. Third, some studies were excluded because of missing data despite contacting authors and pharmaceutical companies (*cf.* Table S1), which may have slightly biased our results. Finally, our results are not extrapolable to children, other conditions than rheumatoid arthritis or exposure exceeding two years.

This study is the largest RCT MA devoted to investigate a putative excess cancer risk in adult rheumatoid arthritis patients exposed to TNF-α antagonists. This study did not find a significant increased risk of cancer during up to two years of treatment. Nonetheless, there was a tendency to an increased rate of NMSC. The risk on long-term exposure was not investigated here but results from observational studies also highlighted the risk of NMSCs, while restriction in the present MA to RCTs during at least 52 weeks lead to a slightly higher estimation of overall cancer risk. Methodologically, mITT analysis may overestimate the treatment effect in case of many drop-out patients in placebo arms. On the contrary, PP analysis regards patients exposed during all the RCT. Nevertheless, in assessing very sparse events and in case of many drop-out patients for inefficacy in the placebo arm, PP analysis underestimates the treatment effect. The truth may be between them. We suggest that both analyses should be conducted when assessing sparse events.

## Supporting Information

Table S1 **Trials excluded at the second step of the selection process.**
(DOC)

Table S2 **Characteristics and references of the 33 trials included in the meta-analysis.**
(DOC)

Table S3 **Malignancies that occurred in randomized controlled trials assessing the five marketed anti-TNF-α in adult rheumatoid arthritis patients.**
(DOCX)

Method S1 **Search equations used for the selection of randomized controlled trials.**
(DOC)
