# Supplementary material for: Cancer Risk of Anti-TNF-α at Recommended Doses in Adult Rheumatoid Arthritis: A Meta-Analysis with Intention to Treat and per Protocol Analyses
Source: PLoS One. 2012 Nov 14;7(11):e48991. doi: 10.1371/journal.pone.0048991 (PMC3498371; doi:10.1371/journal.pone.0048991)
Supplement: Table S1 — Trials excluded at the second step of the selection process. (DOC) [file pone.0048991.s001.doc]

**Table S1** Trials excluded at the second step of the selection process.

| **Trials** | **Anti-TNF-α** | **Number of patients** | **Reasons for exclusion** |
| --- | --- | --- | --- |
| Wong *Ann Rheum Dis* 2009 [1] | IFX | 26 | Safety data not provided |
| Schiff *Ann Rheum Dis* 2008 [2] | IFX | 275 | Not in line with the NDA (infusion at Week 12) |
| Maini *Arthritis Rheum* 1998 [3] | IFX | 103 | Not in line with the NDA (infusion at Week 10) |
| Taylor *Arthritis Rheum* 2006 [4] | IFX | 24 | Non-comparative extension of the trial by Taylor (*Arthritis Rheum* 2004) |
| van Vollenhoven *Lancet* 2009 (SWEFOT) [5] | IFX | 258 | No placebo |
| Haugeberg *Ann Rheum Dis* 2009 [6] | IFX | 20 | Sub-analysis of the trial by Quinn (*Arthritis Rheum* 2005) |
| van der Kooij *Arthritis Rheum* 2009 [7] | IFX | 508 | Sub-analysis of the BeSt study; no placebo |
| Smolen *Ann Rheum Dis* 2009 [8] | IFX | 1049 | Sub-analysis of the ASPIRE study |
| Goekoop-Ruiterman *Arthritis Rheum* 2008 (BeSt) [9] | IFX | 508 | Duplicate with Goekoop-Ruiterman *Arthritis Rheum* 2005; no placebo |
| Durez *Arthritis Rheum* 2007 [10] | IFX | 44 | No placebo |
| Wisłowska *Rheumatol Int* 2007 [11] | IFX | 78 | No randomization |
| Allaart *Clin Exp Rheumatol* 2006 (BeSt) [12] | IFX | 508 | Duplicate with Goekoop-Ruiterman *Arthritis Rheum* 2005; no placebo |
| Smolen *Arthritis Rheum* 2006 [13] | IFX | 1049 | Sub-analysis of the ASPIRE study |
| Lipsky *New Engl J Med* 2000 (ATTRACT) [14] | IFX | 259 | Results at 1 year of the ATTRACT study |
| Maini *Lancet* 1999 (ATTRACT) [15] | IFX | 428 | Results at Week 30 of the ATTRACT study |
| Elliott *Arthritis Rheum*1993 (C0168T07) [16] | IFX | 20 | Dose not in line with the NDA; non-comparative study |
| Elliott *Lancet* 1994 (C0168T09) [17] | IFX | 73 | Dose not in line with the NDA; single infusion |
| C0168T15/17 (phase II), EPAR [18] | IFX | ? | Safety data not provided |
| C0168T18 (phase I), EPAR [18] | IFX | ? | Safety data not provided |
| NCT00006292 [19] | IFX | 60 | Safety data not provided |
| NCT00202852 (Study P04280) [20] | IFX | 143 | Safety data not provided |
| Kaine *J Rheumatol* 2007 [21] | ADA | 226 | Dose not in line with the NDA (first injection, 80 mg) |
| Rau *Scand J Rheumatol* 2004 [22] | ADA | 54 | Dose not in line with the NDA |
| Etude DE001/DE003, EPAR [23] | ADA | 120 | Administration way not in line with the NDA (intravenous) |
| Weisman *Clin Therapeutics* 2003 (*DE005*/DE005X) [24] | ADA | 24 | Administration way not in line with the NDA (intravenous) |
| Schatternkirchner Abstract ACR 1998 (DE004) [25] | ADA | 60 | Dose not in line with the NDA |
| Etude DE010, EPAR [23] | ADA | 54 | Dose not in line with the NDA; single dose |
| van de Putte *Ann Rheum Dis* 2003 (DE007) [26] | ADA | 284 | Dose not in line with the NDA |
| Kavanaugh *Abstract ACR* 2010 (OPTIMA) [27] | ADA | 1032 | Number of malignancies not provided before publication |
| Emery *J Rheumatol* 2009 [28] | ADA | 799 | Sub-analysis of the PREMIER study |
| NCT00234897 (M04-684, HERO) [29] | ADA | 1938 | Safety data not provided |
| Lareskog *Lancet*  2004 [30] | ETN | 503 | Results at Year 1 of the TEMPO study |
| van der Heijde *Arthritis Rheum* 2007 [31] | ETN | 414 | Results at Year 3 of the TEMPO study |
| Emery *Arthritis Rheum*2010 [32] | ETN | 411 | Results at Year 2 of the COMET study |
| Kosinsky *Am J Manag Care* 2002 [33] | ETN | 424 | Sub-analysis of the ERA study |
| Combe *Ann Rheum Dis* 2009 [34] | ETN | 131 | Results at Year 2 of the ES-309 study |
| Keystone *Arthritis Rheum* 2004 [35] | ETN | 420 | Exposure < 12 weeks |
| Sennels *Scand J Rheumatol* 2008 [36] | ETN | 25 | Sub-analysis of the ADORE study, no placebo |
| Marcora *Am J Clin Nutr* 2006 [37] | ETN | 25 | No placebo |
| Kekow *Ann Rheum Dis* 2010 [38] | ETN | 528 | Sub-analysis of the COMET study |
| Hu *J Rheumatol Int* 2009 [39] | ETN | 238 | Exact number of malignancies not provided |
| Zhou *J Clin Pharmacol* 2004 [40] | ETN | 98 | Sub-analysis of the TEMPO study |
| van Riel *Ann Rheum Dis* 2006 (ADORE) [41] | ETN | 315 | No placebo |
| Mathias *Clin Therapeutics* 2000 [42] | ETN | 234 | Sub-analysis of the trial by Moreland (*Ann Int Med* 1999) |
| Moreland *Abstract ACR* 2009 (TEAR) [43] | ETN | 755 | Number of malignancies not provided before publication |
| Sheehy *Abstract ACR* 2008 [44] | ETN | 20 | No placebo |
| 16.0016 (phase II/III), EPAR [45] | ETN | 51 | Paediatric population |
| NCT00445770 [46] | ETN | 550 | Safety data not provided |
| NCT00443950 [47] | ETN | 150 | Safety data not provided |
| Fleischmann *Ann Rheum Dis* 2009 (FAST4WARD) [48] | CTZ | 220 | Dose not in line with the NDA |
| Strand *Arthritis Res Ther* 2009 [49] | CTZ | 982 | Sub-analysis of the RAPID 1 study |
| Choy *Rheumatology* 2002 (CDP870-002) [50] | CTZ | 36 | Single intravenous dose |
| Etude CDP870-004, EPAR [51] | CTZ | 204 | Dose not in line with the NDA |
| Etude NCT00544154 (CDP870-014), EPAR [51] | CTZ | 247 | Dose not in line with the NDA |
| NCT00674362 (CERTAIN) [52] | CTZ | 194 | Exact number of malignancies not provided before publication |
| Kremer *Arthritis Rheum*2010 [53] | GMM | 643 | Administration way not in line with the NDA |
| Visvanathan *J Rheumatol* 2009 [54] | GMM | 171 | Sub-analysis of Kay *Arthritis Rheum* 2008 |
| Fleischmann *Abstract ACR* 2004 (phase I) [55] | GMM | 148 | Dose not in line with the NDA |

Abbreviations: ACR, American College of Rheumatology; BSR, British Society of Rheumatology; ADA, adalimumab; CTZ , certolizumab pegol; EPAR: scientific discussion of the European Public Assessment Report; ETN, etanercept; GMM, golimumab; IFX, infliximab; NDA , New Drug Approval.

**REFERENCES**

1. Wong M, Oakley SP, Young L, Jiang BY, Wierzbicki A, et al. (2009) Infliximab improves vascular stiffness in patients with rheumatoid arthritis. Ann Rheum Dis 68: 1277–1284.

2. Schiff M, Keiserman M, Codding C, Songcharoen S, Berman A, et al. (2008) Efficacy and safety of abatacept or infliximab vs placebo in ATTEST: a phase III, multi-centre, randomised, double-blind, placebo-controlled study in patients with rheumatoid arthritis and an inadequate response to methotrexate. Ann Rheum Dis 67: 1096–1103.

3. Maini RN, Breedveld FC, Kalden JR, Smolen JS, Davis D, et al. (1998) Therapeutic efficacy of multiple intravenous infusions of anti-tumor necrosis factor alpha monoclonal antibody combined with low-dose weekly methotrexate in rheumatoid arthritis. Arthritis Rheum 41: 1552–1563.

4. Taylor PC, Steuer A, Gruber J, McClinton C, Cosgrove DO, et al. (2006) Ultrasonographic and radiographic results from a two-year controlled trial of immediate or one-year-delayed addition of infliximab to ongoing methotrexate therapy in patients with erosive early rheumatoid arthritis. Arthritis Rheum 54: 47–53.

5. van Vollenhoven RF, Ernestam S, Geborek P, Petersson IF, Cöster L, et al. (2009) Addition of infliximab compared with addition of sulfasalazine and hydroxychloroquine to methotrexate in patients with early rheumatoid arthritis (Swefot trial): 1-year results of a randomised trial. Lancet 374: 459–466.

6. Haugeberg G, Conaghan PG, Quinn M, Emery P (2009) Bone loss in patients with active early rheumatoid arthritis: infliximab and methotrexate compared with methotrexate treatment alone. Explorative analysis from a 12-month randomised, double-blind, placebo-controlled study. Ann Rheum Dis 68: 1898–1901.

7. van der Kooij SM, de Vries-Bouwstra JK, Goekoop-Ruiterman YPM, Ewals JAPM, Han KH, et al. (2009) Patient-reported outcomes in a randomized trial comparing four different treatment strategies in recent-onset rheumatoid arthritis. Arthritis Rheum 61: 4–12.

8. Smolen JS, Han C, van der Heijde DMFM, Emery P, Bathon JM, et al. (2009) Radiographic changes in rheumatoid arthritis patients attaining different disease activity states with methotrexate monotherapy and infliximab plus methotrexate: the impacts of remission and tumour necrosis factor blockade. Ann Rheum Dis 68: 823–827.

9. Goekoop-Ruiterman YPM, de Vries-Bouwstra JK, Allaart CF, van Zeben D, Kerstens PJSM, et al. (2005) Clinical and radiographic outcomes of four different treatment strategies in patients with early rheumatoid arthritis (the BeSt study): a randomized, controlled trial. Arthritis Rheum 52: 3381–3390.

10. Durez P, Malghem J, Nzeusseu Toukap A, Depresseux G, Lauwerys BR, et al. (2007) Treatment of early rheumatoid arthritis: a randomized magnetic resonance imaging study comparing the effects of methotrexate alone, methotrexate in combination with infliximab, and methotrexate in combination with intravenous pulse methylprednisolone. Arthritis Rheum 56: 3919–3927.

11. Wisłowska M, Jakubicz D (2007) Preliminary evaluation in rheumatoid arthritis activity in patients treated with TNF-alpha blocker plus methotrexate versus methotrexate or leflunomide alone. Rheumatol Int 27: 641–647.

12. Allaart CF, Goekoop-Ruiterman YPM, de Vries-Bouwstra JK, Breedveld FC, Dijkmans BAC (2006) Aiming at low disease activity in rheumatoid arthritis with initial combination therapy or initial monotherapy strategies: the BeSt study. Clin Exp Rheumatol 24: S–77–82.

13. Smolen JS, Van Der Heijde DMFM, St Clair EW, Emery P, Bathon JM, et al. (2006) Predictors of joint damage in patients with early rheumatoid arthritis treated with high-dose methotrexate with or without concomitant infliximab: results from the ASPIRE trial. Arthritis Rheum 54: 702–710.

14. Maini RN, Breedveld FC, Kalden JR, Smolen JS, Furst D, et al. (2004) Sustained improvement over two years in physical function, structural damage, and signs and symptoms among patients with rheumatoid arthritis treated with infliximab and methotrexate. Arthritis Rheum 50: 1051–1065.

15. Maini R, St Clair EW, Breedveld F, Furst D, Kalden J, et al. (1999) Infliximab (chimeric anti-tumour necrosis factor alpha monoclonal antibody) versus placebo in rheumatoid arthritis patients receiving concomitant methotrexate: a randomised phase III trial. ATTRACT Study Group. Lancet 354: 1932–1939.

16. Elliott MJ, Maini RN, Feldmann M, Long-Fox A, Charles P, et al. (1993) Treatment of rheumatoid arthritis with chimeric monoclonal antibodies to tumor necrosis factor alpha. Arthritis Rheum 36: 1681–1690.

17. Elliott MJ, Maini RN, Feldmann M, Kalden JR, Antoni C, et al. (1994) Randomised double-blind comparison of chimeric monoclonal antibody to tumour necrosis factor alpha (cA2) versus placebo in rheumatoid arthritis. Lancet 344: 1105–1110.

18. European Medicines Agency - Human medicines - Remicade. Available: http://www.ema.europa.eu/ema/index.jsp?curl=pages/medicines/human/medicines/000240/human_med_001023.jsp&murl=menus/medicines/medicines.jsp&mid=WC0b01ac058001d124&jsenabled=true. Accessed 30 May 2011.

19. NCT00006292 ClinicalTrials.gov (n.d.). Available: http://clinicaltrials.gov/ct2/results?term=NCT00006292. Accessed 30 May 2011.

20. A Placebo-Controlled, Double-Blinded, Randomized Trial of Remicade in Korean Patients With Rheumatoid Arthritis Despite Methotrexate (Study P04280). ClinicalTrials.gov. Available: http://clinicaltrials.gov/ct2/show/NCT00202852?term=NCT00202852&rank=1. Accessed 30 May 2011.

21. Kaine JL, Kivitz AJ, Birbara C, Luo AY (2007) Immune responses following administration of influenza and pneumococcal vaccines to patients with rheumatoid arthritis receiving adalimumab. J Rheumatol 34: 272–279.

22. Rau R, Simianer S, van Riel PLCM, van de Putte LBA, Krüger K, et al. (2004) Rapid alleviation of signs and symptoms of rheumatoid arthritis with intravenous or subcutaneous administration of adalimumab in combination with methotrexate. Scand J Rheumatol 33: 145–153.

23. European Medicines Agency - European public assessment reports - Humira. Available: http://www.ema.europa.eu/ema/index.jsp?curl=pages/medicines/human/medicines/000481/human_med_000822.jsp&murl=menus/medicines/medicines.jsp&mid=WC0b01ac058001d125&jsenabled=true. Accessed 30 May 2011.

24. Weisman MH, Moreland LW, Furst DE, Weinblatt ME, Keystone EC, et al. (2003) Efficacy, pharmacokinetic, and safety assessment of adalimumab, a fully human anti-tumor necrosis factor-alpha monoclonal antibody, in adults with rheumatoid arthritis receiving concomitant methotrexate: a pilot study. Clin Ther 25: 1700–1721.

25. Schattenkirchner M, Krüger K, Sander O, Rau R, Kroot E-J, et al. (1998) Efficacy and tolerability of wekly subcutaneous injections of the fully human anti-TNF-antibody D2E7 in aptients with rheumatoid arthritis - Results of a phase I study. Arthritis Rheum 41: S57.

26. van de Putte LBA, Rau R, Breedveld FC, Kalden JR, Malaise MG, et al. (2003) Efficacy and safety of the fully human anti-tumour necrosis factor alpha monoclonal antibody adalimumab (D2E7) in DMARD refractory patients with rheumatoid arthritis: a 12 week, phase II study. Ann Rheum Dis 62: 1168–1177.

27. Kavanaugh A, Fleischmann R, Emery P, Guerette B, Redden L, et al. (2010) Clinical and Functionnal improvements in early RA following treatment with adalimumab plus methotrexate comapred with methotexate monotherapy: 26-weeks results of the OPTIMA trial. Arthritis Rheum 62: 1791.

28. Emery P, Genovese MC, van Vollenhoven R, Sharp JT, Patra K, et al. (2009) Less radiographic progression with adalimumab plus methotrexate versus methotrexate monotherapy across the spectrum of clinical response in early rheumatoid arthritis. J Rheumatol 36: 1429–1441.

29. NCT00234897 ClinicalTrials.gov. Available: http://clinicaltrials.gov/ct2/results?term=NCT00234897+. Accessed 30 May 2011.

30. Klareskog L, van der Heijde D, de Jager JP, Gough A, Kalden J, et al. (2004) Therapeutic effect of the combination of etanercept and methotrexate compared with each treatment alone in patients with rheumatoid arthritis: double-blind randomised controlled trial. Lancet 363: 675–681.

31. van der Heijde D, Klareskog L, Landewé R, Bruyn GAW, Cantagrel A, et al. (2007) Disease remission and sustained halting of radiographic progression with combination etanercept and methotrexate in patients with rheumatoid arthritis. Arthritis Rheum 56: 3928–3939.

32. Emery P, Breedveld F, van der Heijde D, Ferraccioli G, Dougados M, et al. (2010) Two-year clinical and radiographic results with combination etanercept-methotrexate therapy versus monotherapy in early rheumatoid arthritis: a two-year, double-blind, randomized study. Arthritis Rheum 62: 674–682.

33. Kosinski M, Kujawski SC, Martin R, Wanke LA, Buatti MC, et al. (2002) Health-related quality of life in early rheumatoid arthritis: impact of disease and treatment response. Am J Manag Care 8: 231–240.

34. Combe B, Codreanu C, Fiocco U, Gaubitz M, Geusens PP, et al. (2009) Efficacy, safety and patient-reported outcomes of combination etanercept and sulfasalazine versus etanercept alone in patients with rheumatoid arthritis: a double-blind randomised 2-year study. Ann Rheum Dis 68: 1146–1152.

35. Keystone EC, Schiff MH, Kremer JM, Kafka S, Lovy M, et al. (2004) Once-weekly administration of 50 mg etanercept in patients with active rheumatoid arthritis: results of a multicenter, randomized, double-blind, placebo-controlled trial. Arthritis Rheum 50: 353–363.

36. Sennels H, Sørensen S, Ostergaard M, Knudsen L, Hansen M, et al. (2008) Circulating levels of osteopontin, osteoprotegerin, total soluble receptor activator of nuclear factor-kappa B ligand, and high-sensitivity C-reactive protein in patients with active rheumatoid arthritis randomized to etanercept alone or in combination with methotrexate. Scand J Rheumatol 37: 241–247.

37. Marcora SM, Chester KR, Mittal G, Lemmey AB, Maddison PJ (2006) Randomized phase 2 trial of anti-tumor necrosis factor therapy for cachexia in patients with early rheumatoid arthritis. Am J Clin Nutr 84: 1463–1472.

38. Kekow J, Moots RJ, Emery P, Durez P, Koenig A, et al. (2010) Patient-reported outcomes improve with etanercept plus methotrexate in active early rheumatoid arthritis and the improvement is strongly associated with remission: the COMET trial. Ann Rheum Dis 69: 222–225.

39. Hu D, Bao C, Chen S, Gu J, Li Z, et al. (2009) A comparison study of a recombinant tumor necrosis factor receptor:Fc fusion protein (rhTNFR:Fc) and methotrexate in treatment of patients with active rheumatoid arthritis in China. Rheumatol Int 29: 297–303.

40. Zhou H, Mayer PR, Wajdula J, Fatenejad S (2004) Unaltered etanercept pharmacokinetics with concurrent methotrexate in patients with rheumatoid arthritis. J Clin Pharmacol 44: 1235–1243.

41. van Riel PLCM, Taggart AJ, Sany J, Gaubitz M, Nab HW, et al. (2006) Efficacy and safety of combination etanercept and methotrexate versus etanercept alone in patients with rheumatoid arthritis with an inadequate response to methotrexate: the ADORE study. Ann Rheum Dis 65: 1478–1483.

42. Mathias SD, Colwell HH, Miller DP, Moreland LW, Buatti M, et al. (2000) Health-related quality of life and functional status of patients with rheumatoid arthritis randomly assigned to receive etanercept or placebo. Clin Ther 22: 128–139.

43. Moreland LW, O’Dell JR, Paulus HE, Curtis JR, Bridges Jr S, et al. (2009) TEAR: Treatment of early aggressive RA: A randomized, double-blind, 2 year trial comparing immediate triple DMARD versus MTX plus etanercept step-up from initial MTX monotherapy. Arthritis and Rheumatism 60: 1895.

44. Sheehy C, Murphy E, Duffy T, Barry M (2008) Remission induction with etanercept and methotrexate in very early rheumatoid arthritis with sustained remission after etanercept withdrawal. Arthritis Rheum 58: 918.

45. European Medicines Agency - Enbrel. Available: http://www.ema.europa.eu/ema/index.jsp?curl=pages/medicines/human/medicines/000262/human_med_000764.jsp&murl=menus/medicines/medicines.jsp&jsenabled=true. Accessed 30 May 2011.

46. NCT00445770 - ClinicalTrials.gov. Available: http://clinicaltrials.gov/ct2/results?term=NCT00445770+. Accessed 30 May 2011.

47. NCT00443950 - ClinicalTrials.gov. Available: http://clinicaltrials.gov/ct2/results?term=NCT00443950. Accessed 30 May 2011.

48. Fleischmann R, Vencovsky J, van Vollenhoven RF, Borenstein D, Box J, et al. (2009) Efficacy and safety of certolizumab pegol monotherapy every 4 weeks in patients with rheumatoid arthritis failing previous disease-modifying antirheumatic therapy: the FAST4WARD study. Ann Rheum Dis 68: 805–811.

49. Strand V, Mease P, Burmester GR, Nikaï E, Coteur G, et al. (2009) Rapid and sustained improvements in health-related quality of life, fatigue, and other patient-reported outcomes in rheumatoid arthritis patients treated with certolizumab pegol plus methotrexate over 1 year: results from the RAPID 1 randomized controlled trial. Arthritis Res Ther 11: R170.

50. Choy EHS, Hazleman B, Smith M, Moss K, Lisi L, et al. (2002) Efficacy of a novel PEGylated humanized anti-TNF fragment (CDP870) in patients with rheumatoid arthritis: a phase II double-blinded, randomized, dose-escalating trial. Rheumatology (Oxford) 41: 1133–1137.

51. European Medicines Agency - Human medicines - Cimzia. Available: http://www.ema.europa.eu/ema/index.jsp?curl=pages/medicines/human/medicines/001037/human_med_001294.jsp&mid=WC0b01ac058001d124&murl=menus/medicines/medicines.jsp&jsenabled=true. Accessed 30 May 2011.

52. NCT00674362 - ClinicalTrials.gov. Available: http://clinicaltrials.gov/ct2/results?term=NCT00674362. Accessed 30 May 2011.

53. Kremer J, Ritchlin C, Mendelsohn A, Baker D, Kim L, et al. (2010) Golimumab, a new human anti-tumor necrosis factor alpha antibody, administered intravenously in patients with active rheumatoid arthritis: Forty-eight-week efficacy and safety results of a phase III randomized, double-blind, placebo-controlled study. Arthritis Rheum 62: 917–928.

54. Visvanathan S, Wagner C, Rojas J, Kay J, Dasgupta B, et al. (2009) E-selectin, interleukin 18, serum amyloid a, and matrix metalloproteinase 9 are associated with clinical response to golimumab plus methotrexate in patients with active rheumatoid arthritis despite methotrexate therapy. J Rheumatol 36: 1371–1379.

55. Fleischmann R, Cohen S, Caldwell JR, Offenberg HL, Bouman-Thio E, et al. (2004) Phase I studies evaluating the safety, pharmacokinetics, and pharmacodynamics of intravenous and subcutaneous administration of a fully human monoclonal antibody to human TNF-a (CNTO 148) in rheumaotid arthritis patients. Arthritis Rheum 50: 178.
