# Supplementary material for: Cancer Risk of Anti-TNF-α at Recommended Doses in Adult Rheumatoid Arthritis: A Meta-Analysis with Intention to Treat and per Protocol Analyses
Source: PLoS One. 2012 Nov 14;7(11):e48991. doi: 10.1371/journal.pone.0048991 (PMC3498371; doi:10.1371/journal.pone.0048991)
Supplement: Table S2 — Characteristics and references of the 33 trials included in the meta-analysis. (DOC) [file pone.0048991.s002.doc]

**Table S2** Characteristics and references of the 33 trials included in the meta-analysis.

| **Trial** | **Condition** | **Date of safety evaluation (weeks)** | **Treatment groups** | | | **Comparator groups** | | |
| --- | --- | --- | --- | --- | --- | --- | --- | --- |
|  |  |  | **Arms** | **Number of patients (mITT)** | **Number of patients (PP)** | **Arms** | **Number of patients (mITT)** | **Number of patients (PP)** |
| Maini, 2004 | Active RA despite MTX therapy | 102 | IFX 3mg/kg/8W + MTX* | 86 | 48 | Placebo + MTX | 88 | 15 |
|  |  |  | IFX 10mg/kg/8W + MTX | 87 | 62 |  |  |  |
|  |  |  | IFX 3mg/kg/4W + MTX | 86 | 47 |  |  |  |
|  |  |  | IFX 10mg/kg/4W + MTX | 81 | 54 |  |  |  |
| St Clair, 2004 | Active early RA<3 years without previous MTX therapy | 54 | IFX 3mg/kg/8W + MTX* | 373 | 323 | Placebo + MTX | 298 | 245 |
|  |  |  | IFX 6mg/kg/8W + MTX* | 378 | 329 |  |  |  |
| Taylor, 2004 | Active early RA<3 years on MTX therapy | 54 | IFX 5mg/kg/8W + MTX* | 12 | 12 | Placebo + MTX | 12 | 11 |
| Quinn, 2005 | Active early RA<1 year without previous DMARD therapy | 54 | IFX 3mg/kg/8W + MTX* | 10 | 9 | Placebo + MTX | 10 | 10 |
| Abe, 2006 | Active RA despite MTX therapy | 14 | IFX 3mg/kg/8W + MTX* | 49 | 48 | Placebo + MTX | 47 | 42 |
|  |  |  | IFX 10mg/kg/8W + MTX | 51 | 47 |  |  |  |
| Westhovens, 2006 | Active RA despite MTX therapy | 54 | IFX 3mg/kg/8W + MTX* | 360 | 306 | Placebo + MTX | 361 | 303 |
|  |  |  | IFX 10mg/kg/8W + MTX | 361 | 303 |  |  |  |
| Leirisalo-Repo, 2009 | Active early RA ≤1 year | 26 | IFX 3mg/kg/8W + COMBI* | 50 | 46 | Placebo + COMBI | 49 | 45 |
| Moreland, 1997 | Active RA despite DMARD therapy | 12 | ETN 0.25mg/m² x 2/W | 46 | 28 | Placebo | 44 | 23 |
|  |  |  | ETN 2mg/m² x 2/W | 46 | 36 |  |  |  |
| Weinblatt, 1999 | Active RA despite MTX therapy | 24 | ETN 25mg x 2/W + MTX* | 59 | 57 | Placebo + MTX | 30 | 24 |
|  |  |  | ETN 16mg/m² x 2/W* | 44 | 41 |  |  |  |
| Moreland, 1999 | Active RA despite ≥ 1 DMARD | 26 | ETN 10mg x 2/W | 76 | 52 | Placebo | 80 | 26 |
|  |  |  | ETN 25mg x 2/W* | 78 | 59 |  |  |  |
| Ericson, 1999 | Active RA despite DMARD therapy | 12 | ETN 10mg /W | 122 | 114 | Placebo | 105 | 85 |
|  |  |  | ETN 10mg x 2/W | 110 | 112 |  |  |  |
|  |  |  | ETN 25mg /W | 111 | 101 |  |  |  |
|  |  |  | ETN 25mg x 2/W* | 111 | 105 |  |  |  |
| Bathon, 2000 | Active early RA ≤ 3 years without previous MTX therapy | 52 | ETN 10mg x 2/W + Placebo | 208 | 168 | Placebo + MTX | 217 | 174 |
|  |  |  | ETN 25mg x 2/W + Placebo* | 207 | 179 |  |  |  |
| Lan, 2004 | Active RA ≥ 1 year | 12 | ETN 25mg x 2/W + MTX* | 29 | 27 | Placebo + MTX | 29 | 27 |
| van der Heijde, 2006 | Active RA despite ≥ 1 DMARD excluding MTX | 104 | ETN 25mg x 2/W+ MTX* | 231 | 169 | Placebo + MTX | 228 | 121 |
|  |  |  | ETN 25mg x 2/W+ Placebo* | 223 | 142 |  |  |  |
| Weisman, 2007 | Active RA | 20 | ETN 25mg x 2/W* | 266 | 231 | Placebo | 269 | 197 |
| Combe, 2009 | Active RA on SSZ for ≥ 4 months | 104 | ETN 25mg x 2/W + Placebo* | 103 | 67 | Placebo + SSZ | 50 | 16 |
|  |  |  | ETN 25mg x 2/W + SSZ^†^ | 101 | 77 |  |  |  |
| Emery, 2010 | Active early RA < 2 years without previous MTX therapy | 104 | ETN 50mg/W+ MTX* | 163 | 108 | Placebo + MTX | 178 | 83 |
| Weinblatt, 2003 | Active RA despite MTX and having failed to ≥ 1 other DMARD | 24 | ADA 20mg eow + MTX | 69 | 42 | Placebo + MTX | 62 | 18 |
|  |  |  | ADA 40mg eow + MTX* | 67 | 50 |  |  |  |
|  |  |  | ADA 80mg eow + MTX | 73 | 55 |  |  |  |
| Furst, 2003 | Active RA | 24 | ADA 40mg eow + DMARD* | 318 | 294 | Placebo + DMARD | 318 | 288 |
| van de Putte, 2004 | Active RA having failed to ≥ 1 DMARD | 26 | ADA 20mg/W | 112 | 79 | Placebo | 110 | 49 |
|  |  |  | ADA 20mg eow | 106 | 69 |  |  |  |
|  |  |  | ADA 40mg/W | 103 | 89 |  |  |  |
|  |  |  | ADA 40mg eow* | 113 | 83 |  |  |  |
| Keystone, 2004 | Active RA despite MTX therapy | 52 | ADA 40mg eow + MTX* | 207 | 164 | Placebo + MTX | 200 | 141 |
|  |  |  | ADA 20mg/W + MTX | 212 | 171 |  |  |  |
| Breedveld, 2006 | Active early RA < 3 years without previous MTX or > 2 other DMARDs | 104 | ADA 40mg eow + MTX* | 268 | 205 | Placebo + MTX | 257 | 173 |
|  |  |  | ADA 40mg eow + Placebo* | 274 | 171 |  |  | 173 |
| Kim, 2007 | Active RA despite MTX and having failed to ≥ 1 other DMARD | 24 | ADA 40mg eow + MTX* | 65 | 55 | Placebo + MTX | 63 | 40 |
| Miyasaka, 2008 | Active RA despite ≥ 1 DMARD | 24 | ADA 20mg eow | 87 | 54 | Placebo | 87 | 38 |
|  |  |  | ADA 40mg eow* | 91 | 59 |  |  |  |
|  |  |  | ADA 80mg eow | 87 | 65 |  |  |  |
| Bejarano, 2008 | Active early RA ≤ 2 years without previous MTX therapy | 56 | ADA 40mg eow + MTX* | 75 | 50 | Placebo + MTX | 73 | 36 |
| Chen, 2009 | Active RA ≥ 1 year | 12 | ADA 40mg eow + MTX* | 35 | 32 | Placebo + MTX | 12 | 12 |
| Kay, 2008 | Active RA despite MTX therapy | 20 | GMM 50mg/4W + MTX* | 35 | 31 | Placebo + MTX | 35 | 29 |
|  |  |  | GMM 50mg/2W + MTX | 34 | 29 |  |  |  |
|  |  |  | GMM 100mg/4W + MTX | 34 | 30 |  |  |  |
|  |  |  | GMM 100mg/2W + MTX | 34 | 32 |  |  |  |
| Keystone, 2009 | Active RA despite MTX therapy | 24 | GMM 50mg/4W + MTX* | 89 | 72 | Placebo + MTX | 133 | 85 |
|  |  |  | GMM 100mg/4W + Placebo | 133 | 93 |  |  |  |
|  |  |  | GMM 100mg/4W + MTX | 89 | 83 |  |  |  |
| Emery, 2009 | Active RA without previous MTX therapy | 24 | GMM 50mg/4W + MTX* | 158 | 151 | Placebo + MTX | 160 | 152 |
|  |  |  | GMM 100mg/4W + Placebo | 157 | 148 |  |  |  |
|  |  |  | GMM 100mg/4W + MTX | 159 | 152 |  |  |  |
| Tanaka, 2010 | Active RA despite MTX therapy | 24 | GMM 50mg/4W + MTX* | 86 | 76 | Placebo + MTX | 88 | 58 |
|  |  |  | GMM 100mg/4W + MTX | 87 | 80 |  |  |  |
| Takeuchi, 2010 | Active RA despite DMARD | 16 | GMM 50mg/4W* | 101 | 96 | Placebo | 106 | 92 |
|  |  |  | GMM 100mg/4W | 102 | 100 |  |  |  |
| Keystone, 2008 | Active RA despite MTX therapy | 52 | CTZ 200mg eow + MTX* | 383 | 252 | Placebo + MTX | 192 | 37 |
|  |  |  | CTZ 400mg eow + MTX | 375 | 263 |  |  |  |
| Smolen, 2009 | Active RA despite MTX therapy | 24 | CTZ 200mg eow + MTX* | 242 | 171 | Placebo + MTX | 125 | 16 |
|  |  |  | CTZ 400mg eow + MTX | 244 | 180 |  |  |  |

Abbreviations: ADA, adalimumab; COMBI, combination treatment (methotrexate, sulfasalazine and hydroxychloroquine); CTZ, certolizumabpegol; DMARD, Disease-modifying antirheumatic drug; eow, every other week; ETN, etanercept; GMM, golimumab; IFX, infliximab; mITT, modified intention to treat analysis; MTX, methotrexate; PP, per protocol analysis; SSZ, sulfasalazine.

* Dose in line with the New Drug Approval.

^†^Not included in the analyses (non in line with the New Drug Approval).

**References:**

1. Maini RN, Breedveld FC, Kalden JR, Smolen JS, Furst D, et al. (2004) Sustained improvement over two years in physical function, structural damage, and signs and symptoms among patients with rheumatoid arthritis treated with infliximab and methotrexate. Arthritis Rheum 50: 1051-65.

2. St Clair EW, van der Heijde D, Smolen JS, Maini RN, Bathon JM, et al. (2004) Combination of infliximab and methotrexate therapy for early rheumatoid arthritis: a randomized, controlled trial. Arthritis Rheum 50: 3432-43.

3. Taylor PC, Steuer A, Gruber J, Cosgrove DO, Blomley MJK, et al. (2004) Comparison of ultrasonographic assessment of synovitis and joint vascularity with radiographic evaluation in a randomized, placebo-controlled study of infliximab therapy in early rheumatoid arthritis. Arthritis Rheum 50: 1107-16.

4. Quinn MA, Conaghan PG, O’Connor PJ, Karim Z, Greenstein A, et al. (2005) Very early treatment with infliximab in addition to methotrexate in early, poor-prognosis rheumatoid arthritis reduces magnetic resonance imaging evidence of synovitis and damage, with sustained benefit after infliximab withdrawal: results from a twelve-month randomized, double-blind, placebo-controlled trial. Arthritis Rheum 52: 27-35.

5. Abe T, Takeuchi T, Miyasaka N, Hashimoto H, Kondo H, et al. (2006) A multicenter, double-blind, randomized, placebo controlled trial of infliximab combined with low dose methotrexate in Japanese patients with rheumatoid arthritis. J Rheumatol 33: 37-44.

6. Westhovens R, Yocum D, Han J, Berman A, Strusberg I, et al. (2006) The safety of infliximab, combined with background treatments, among patients with rheumatoid arthritis and various comorbidities: a large, randomized, placebo-controlled trial. Arthritis Rheum 54: 1075-86.

7. Leirisalo-Repo M, Kautiainen H, Laasolen L. (2009) A randomized double-blind placebo-controlled study on addition of 6-months induciton therapy with. Rheumatology 48(Suppl. 1): I11-I12.

8. Moreland LW, Baumgartner SW, Schiff MH, Tindall EA, Fleischmann RM, et al. (1997) Treatment of rheumatoid arthritis with a recombinant human tumor necrosis factor receptor (p75)-Fc fusion protein. N Engl J Med 337: 141-7.

9. Weinblatt ME, Kremer JM, Bankhurst AD, Bulpitt KJ, Fleischmann RM, et al. (1999) A trial of etanercept, a recombinant tumor necrosis factor receptor:Fc fusion protein, in patients with rheumatoid arthritis receiving methotrexate. N Engl J Med 340: 253-9.

10. Moreland LW, Schiff MH, Baumgartner SW, Tindall EA, Fleischmann RM, et al. (1999) Etanercept therapy in rheumatoid arthritis. A randomized, controlled trial. Ann Intern Med 130: 478-86.

11. The European Etanercept Investigators group, Ericson M, Wadjula J. (1999) A double-blind, placebo-controlled study of the efficacy and safety of four different doses of etanercept in patients with rheumatoid arthritis. Arthritis Rheum 42(Suppl. 10): 82.

12. Bathon JM, Martin RW, Fleischmann RM, Tesser JR, Schiff MH, et al. (2000) A comparison of etanercept and methotrexate in patients with early rheumatoid arthritis. N Engl J Med 343: 1586-93.

13. Lan J-L, Chou S-J, Chen D-Y, Chen Y-H, Hsieh T-Y, et al. (2004) A comparative study of etanercept plus methotrexate and methotrexate alone in Taiwanese patients with active rheumatoid arthritis: a 12-week, double-blind, randomized, placebo-controlled study. J Formos Med Assoc 103: 618-23.

14. van der Heijde D, Klareskog L, Rodriguez-Valverde V, Codreanu C, Bolosiu H, et al. (2006) Comparison of etanercept and methotrexate, alone and combined, in the treatment of rheumatoid arthritis: two-year clinical and radiographic results from the TEMPO study, a double-blind, randomized trial. Arthritis Rheum 54: 1063-74.

15. Weisman MH, Paulus HE, Burch FX, Kivitz AJ, Fierer J, et al. (2007) A placebo-controlled, randomized, double-blinded study evaluating the safety of etanercept in patients with rheumatoid arthritis and concomitant comorbid diseases. Rheumatology 46: 1122-5.

16. Combe B, Codreanu C, Fiocco U, Gaubitz M, Geusens PP, et al. (2009) Efficacy, safety and patient-reported outcomes of combination etanercept and sulfasalazine versus etanercept alone in patients with rheumatoid arthritis: a double-blind randomised 2-year study. Ann Rheum Dis 68: 1146-52.

17. Emery P, Breedveld F, van der Heijde D, Ferraccioli G, Dougados M, et al. (2010) Two-year clinical and radiographic results with combination etanercept-methotrexate therapy versus monotherapy in early rheumatoid arthritis: a two-year, double-blind, randomized study. Arthritis Rheum 62: 674-82.

18. Weinblatt ME, Keystone EC, Furst DE, Moreland LW, Weisman MH, et al. (2003) Adalimumab, a fully human anti-tumor necrosis factor alpha monoclonal antibody, for the treatment of rheumatoid arthritis in patients taking concomitant methotrexate: the ARMADA trial. Arthritis Rheum 48: 35-45.

19. Furst DE, Schiff MH, Fleischmann RM, Strand V, Birbara CA, et al. (2003) Adalimumab, a fully human anti tumor necrosis factor-alpha monoclonal antibody, and concomitant standard antirheumatic therapy for the treatment of rheumatoid arthritis: results of STAR (Safety Trial of Adalimumab in Rheumatoid Arthritis. J Rheumatol 30: 2563-71.

20. van de Putte LB, Atkins C, Malaise M, Sany J, Russell AS, et al. (2004) Efficacy and safety of adalimumab as monotherapy in patients with rheumatoid arthritis for whom previous disease modifying antirheumatic drug treatment has failed. Ann Rheum Dis 63: 508-16.

21. Keystone EC, Kavanaugh AF, Sharp JT, Tannenbaum H, Hua Y, et al. (2004) Radiographic, clinical, and functional outcomes of treatment with adalimumab (a human anti-tumor necrosis factor monoclonal antibody) in patients with active rheumatoid arthritis receiving concomitant methotrexate therapy: a randomized, placebo-controlled, 52-week trial. Arthritis Rheum 50: 1400-11.

22. Breedveld FC, Weisman MH, Kavanaugh AF, Cohen SB, Pavelka K, et al. (2006) The PREMIER study: A multicenter, randomized, double-blind clinical trial of combination therapy with adalimumab plus methotrexate versus methotrexate alone or adalimumab alone in patients with early, aggressive rheumatoid arthritis who had not had previous methotrexate treatment. Arthritis Rheum 54: 26-37.

23. Kim H-Y, Lee S-K, Song YW, Yoo D-H, Koh E-M, et al. (2007) A randomized, double-blind, placebo-controlled, phase III study of the human anti-tumor necrosis factor antibody adalimumab administered as subcutaneous injections in Korean rheumatoid arthritis patients treated with methotrexate. APLAR J Rheumatol 10: 9-16.

24. Miyasaka N. (2008) Clinical investigation in highly disease-affected rheumatoid arthritis patients in Japan with adalimumab applying standard and general evaluation: the CHANGE study. Mod Rheumatol 18: 252-62.

25. Bejarano V, Quinn M, Conaghan PG, Reece R, Keenan A-M, et al. (2008) Effect of the early use of the anti-tumor necrosis factor adalimumab on the prevention of job loss in patients with early rheumatoid arthritis. Arthritis Rheum 59: 1467-74.

26. Chen D-Y, Chou S-J, Hsieh T-Y, Chen Y-H, Chen H-H, et al. (2009) Randomized, double-blind, placebo-controlled, comparative study of human anti-TNF antibody adalimumab in combination with methotrexate and methotrexate alone in Taiwanese patients with active rheumatoid arthritis. J Formos Med Assoc 108: 310-9.

27. Kay J, Matteson EL, Dasgupta B, Nash P, Durez P, et al. (2008) Golimumab in patients with active rheumatoid arthritis despite treatment with methotrexate: a randomized, double-blind, placebo-controlled, dose-ranging study. Arthritis Rheum 58: 964-75.

28. Keystone EC, Genovese MC, Klareskog L, Hsia EC, Hall ST, et al. (2009) Golimumab, a human antibody to tumour necrosis factor α given by monthly subcutaneous injections, in active rheumatoid arthritis despite methotrexate therapy: the GO-FORWARD Study. Ann Rheum Dis 68: 789-96.

29. Emery P, Fleischmann RM, Moreland LW, Hsia EC, Strusberg I, et al. (2009) Golimumab, a human anti-tumor necrosis factor alpha monoclonal antibody, injected subcutaneously every four weeks in methotrexate-naive patients with active rheumatoid arthritis: twenty-four-week results of a phase III, multicenter, randomized, double-blind, placebo-controlled study of golimumab before methotrexate as first-line therapy for early-onset rheumatoid arthritis. Arthritis Rheum 60: 2272-83.

30. Tanaka Y, Harigai M, Takeuchi T, Yamanaka H, Ishiguro N, et al. (2010) Golimumab, a human anti-TNF monoclonal antibody administered subcutaneously every four weeks in patients with active rheumatoid arthritis despite methotrexate therapy: 24-week results of clinical and radiographic assessments. Arthritis Rheum 62(Supl. 10): 1815.

31. Takeuchi T, Harigai M, Tanaka Y, Yamanaka H, Ishiguro N, et al. (2010) Golimumab, a human anti-TNF monoclonal antibody administered subcutaneously every four weeks as monotherapy in patients with active rheumatoid arthritis despite DMARD therapy: 24-week results of clinical and radiographic assessments. Arthritis Rheum 62(Suppl. 10): 1814.

32. Keystone E, Heijde D van der, Mason D, Landewé R, Vollenhoven RV, et al. (2008) Certolizumab pegol plus methotrexate is significantly more effective than placebo plus methotrexate in active rheumatoid arthritis: findings of a fifty-two-week, phase III, multicenter, randomized, double-blind, placebo-controlled, parallel-group study. Arthritis Rheum 58: 3319-29.

33. Smolen J, Landewé RB, Mease P, Brzezicki J, Mason D, et al. (2009) Efficacy and safety of certolizumab pegol plus methotrexate in active rheumatoid arthritis: the RAPID 2 study. A randomised controlled trial. Ann Rheum Dis 68: 797-804.
