# Supplementary material for: Cancer Risk of Anti-TNF-α at Recommended Doses in Adult Rheumatoid Arthritis: A Meta-Analysis with Intention to Treat and per Protocol Analyses
Source: PLoS One. 2012 Nov 14;7(11):e48991. doi: 10.1371/journal.pone.0048991 (PMC3498371; doi:10.1371/journal.pone.0048991)
Supplement: Table S3 — Malignancies that occurred in randomized controlled trials assessing the five marketed anti-TNF-α in adult rheumatoid arthritis patients. (DOCX) [file pone.0048991.s003.docx]

**Table S3** Malignancies that occurred in randomized controlled trials assessing the five marketed anti-TNF-α in adult rheumatoid arthritis patients.

| **Trial** | **Anti-TNF-α groups** | | **Comparator groups** | |
| --- | --- | --- | --- | --- |
|  | **Arms** | **Cancers** | **Arms** | **Cancers** |
| Maini, 2004 | IFX 3mg/kg/8W + MTX* | 1 melanoma | Placebo + MTX | 1 NMSC |
|  | IFX 10mg/kg/8W + MTX | 2 NMSC, 1 rectal carcinoma |  |  |
|  | IFX 3mg/kg/4W + MTX | 0 |  |  |
|  | IFX 10mg/kg/4W + MTX | 3 NMSC, 1 melanoma, 1 lymphoma, 1 breast cancer |  |  |
| St Clair, 2004 | IFX 3mg/kg/8W + MTX* | 0 | Placebo + MTX | 0 |
|  | IFX 6mg/kg/8W + MTX* | 1 pancreatic cancer, 1 endometrial cancer, 1 colon adenocarcinoma, 1 acute myeloid leukaemia |  |  |
| Taylor, 2004 | IFX 5mg/kg/8W + MTX* | 0 | Placebo + MTX | 0 |
| Quinn, 2005 | IFX 3mg/kg/8W + MTX* | 0 | Placebo + MTX | 0 |
| Abe, 2006 | IFX 3mg/kg/8W + MTX* | 0 | Placebo + MTX | 0 |
|  | IFX 10mg/kg/8W + MTX | 0 |  |  |
| Westhovens, 2006 | IFX 3mg/kg/8W + MTX* | 2 ovarian carcinomas, 1 non-Hodgkin’s lymphoma, 1 pancreatic cancer, 1 colon carcinoma | Placebo + MTX | 1 renal cell carcinoma, 1 sarcoma |
|  | IFX 10mg/kg/8W + MTX | 2 lung carcinomas, 5 skin cancers ^‡^ |  |  |
| Lerisalo-Repo, 2009 | IFX 3mg/kg/8W + COMBI* | 0 | Placebo + COMBI | 0 |
| Moreland, 1997 | ETN 0.25mg/m² x 2/W | 0 | Placebo | 0 |
|  | ETN 2mg/m² x 2/W | 0 |  |  |
|  | ETN 16mg/m² x 2/W* | 0 |  |  |
| Weinblatt, 1999 | ETN 25mg x 2/W + MTX* | 0 | Placebo + MTX | 0 |
| Moreland, 1999 | ETN 10mg x 2/W | 0 | Placebo | 0 |
|  | ETN 25mg x 2/W* | 0 |  |  |
| Ericson, 1999 | ETN 10mg /W | 1 breast cancer | Placebo | 0 |
|  | ETN 10mg x 2/W | 1 large granular lymphocyte monoclonal leukaemia |  |  |
|  | ETN 25mg /W | 0 |  |  |
|  | ETN 25mg x 2/W* | 0 |  |  |
| Bathon, 2000 | ETN 10mg x 2/W + Placebo | 1 breast cancer, 1 lung cancer | Placebo + MTX | 1 colon cancer, 1 bladder cancer |
|  | ETN 25mg x 2/W + Placebo* | 1 lung cancer, 1 prostate cancer, 1 Hodgkin’s lymphoma |  |  |
| Lan, 2004 | ETN 25mg x 2/W + MTX | 0 | Placebo + MTX | 0 |
| van der Heijde, 2006 | ETN 25mg x 2/W+ MTX* | 2 gastrointestinal cancers, 1 lung cancer, 1 NMSC | Placebo + MTX | 1 breast cancer, 1 NMSC |
|  | ETN 25mg x 2/W+ Placebo* | 2 NMSC, 1 melanoma, 1 breast cancer, 1 rectal cancer |  |  |
| Weisman, 2007 | ETN 25mg x 2/W* | 2 skin cancers | Placebo | 1 skin cancer, 1 lung cancer, 1 carcinoma not otherwise specified |
| Combe, 2009 | ETN 25mg x 2/W + Placebo* | 1 NMSC, 1 myelocytic leukaemia after myelodysplastic syndrome | Placebo + SSZ | 0 |
|  | ETN 25mg x 2/W + SSZ ^†^ | 0 |  |  |
| Emery, 2010 | ETN 50mg/W+ MTX* | 3 NMSC, 1 chronic lymphocytic leukaemia | Placebo + MTX | 3 breast cancers, 1 prostate cancer, 1 pancreas cancer, 1 lung cancer, 1 NMSC |
| Weinblatt, 2003 | ADA 20mg eow + MTX | 0 | Placebo + MTX | 0 |
|  | ADA 40mg eow + MTX* | 0 |  |  |
|  | ADA 80mg eow + MTX | 1 colon adenocarcinoma |  |  |
| Furst, 2003 | ADA 40mg eow + DMARD | 3 NMSC, 1 T cell lymphoma | Placebo + DMARD | 0 |
| van de Putte, 2004 | ADA 20mg/W | 0 | Placebo | 1 NMSC |
|  | ADA 20mg eow | 1 NMSC |  |  |
|  | ADA 40mg/W | 1 gastrointestinal adenocarcinoma |  |  |
|  | ADA 40mg eow* | 1 NMSC, 1 cholangiocarcinoma |  |  |
| Keystone, 2004 | ADA 40mg eow + MTX* | 3 NMSC, 1 breast cancer, 1 gastrointestinal adenocarcinoma | Placebo + MTX | 1 NMSC |
|  | ADA 20mg/W + MTX | 1 breast cancer, 1 seminoma, 1 B cell lymphoma |  |  |
| Breedveld, 2006 | ADA 40mg eow + MTX* | 1 ovarian cancer, 1 prostate cancer | Placebo + MTX | 1 prostate, 1 breast cancer, 1 melanoma, 1 lymphoma |
|  | ADA 40mg eow + Placebo* | 1 breast cancer, 1 colon cancer, 1 cancer with unknown primary site, 1 multiple myeloma |  |  |
| Kim, 2007 | ADA 40mg eow + MTX* | 0 | Placebo + MTX | 0 |
| Miyasaka, 2008 | ADA 20mg eow | 0 | Placebo | 2 cancers not otherwise specified |
|  | ADA 40mg eow* | 0 |  |  |
|  | ADA 80mg eow | 0 |  |  |
| Bejarano, 2008 | ADA 40mg eow + MTX* | 0 | Placebo + MTX | 0 |
| Chen, 2009 | ADA 40mg eow + MTX* | 0 | Placebo + MTX | 0 |
| Kay, 2008 | GMM 50mg/4W + MTX* | 0 | Placebo + MTX | 0 |
|  | GMM 50mg/2W + MTX | 1 lung cancer |  |  |
|  | GMM 100mg/4W + MTX | 1 NMSC |  |  |
|  | GMM 100mg/2W + MTX | 0 |  |  |
| Keystone, 2009 | GMM 50mg/4W + MTX* | 0 | Placebo + MTX | 1 NMSC |
|  | GMM 100mg/4W + Placebo | 2 NMSC |  |  |
|  | GMM 100mg/4W + MTX | 1 breast cancer |  |  |
| Emery, 2009 | GMM 50mg/4W + MTX* | 1 breast cancer | Placebo + MTX | 1 breast cancer, 1 NMSC |
|  | GMM 100mg/4W + Placebo | 0 |  |  |
|  | GMM 100mg/4W + MTX | 1 Hodgkin’s lymphoma |  |  |
| Tanaka, 2010 | GMM 50mg/4W + MTX* | 1 bone neoplasm | Placebo + MTX | 0 |
|  | GMM 100mg/4W + MTX | 0 |  |  |
| Takeuchi, 2010 | GMM 50mg/4W* | 0 | Placebo | 0 |
|  | GMM 100mg/4W | 0 |  |  |
| Keystone, 2008 | CTZ 200mg eow + MTX* | 3 NMSC, 1 adrenal adenoma, 1 hepatic cancer, 1 oesophageal carcinoma, 1 uterine cancer | Placebo + MTX | 1 thyroid cancer |
|  | CTZ 400mg eow + MTX | 2 NMSC, 1 marginal-zone B celllymphoma, 1 papilloma |  |  |
| Smolen, 2009 | CTZ 200mg eow + MTX* | 1 testicular cancer | Placebo + MTX | 1 bladder cancer |
|  | CTZ 400mg eow + MTX | 1 colon cancer |  |  |

Abbreviations: ADA, adalimumab; COMBI, combination treatment (methotrexate, sulfasalazine and hydroxychloroquine); CTZ, certolizumab pegol; DMARD, Disease-modifying antirheumatic drug; eow, every other week; ETN, etanercept; GMM, golimumab; IFX, infliximab; MTX, methotrexate; NMSC, non-melanoma skin cancer; SSZ, sulfasalazine.

* Dose in line with the New Drug Approval.

^†^ Not included in the analyses (non in line with the New Drug Approval).

^‡^We may have overestimated the number of skin cancers that occurred in the 10mg/kg group during the first 54 weeks of the trial. Indeed, data are unclear in the paper and we could not obtain more precise data.
