# Supplementary material for: Cancer Risk of Anti-TNF-α at Recommended Doses in Adult Rheumatoid Arthritis: A Meta-Analysis with Intention to Treat and per Protocol Analyses
Source: PLoS One. 2012 Nov 14;7(11):e48991. doi: 10.1371/journal.pone.0048991 (PMC3498371; doi:10.1371/journal.pone.0048991)
Supplement: Method S1 — Search equations used for the selection of randomized controlled trials. (DOC) [file pone.0048991.s004.doc]

**Method S1** Search equations used for the selection of randomized controlled trials.

1. In MEDLINE, search was performed with MeSH terms until 31 December 2010:

#1 ("Arthritis, Rheumatoid"[Mesh]) AND ("infliximab" [Supplementary Concept])

#2 ("Arthritis, Rheumatoid"[Mesh]) AND ("TNFR-Fc fusion protein" [Supplementary Concept])

#3 ("Arthritis, Rheumatoid"[Mesh]) AND ("adalimumab" [Supplementary Concept])

#4 ("Arthritis, Rheumatoid"[Mesh]) AND ("golimumab" [Supplementary Concept])

#5 ("Arthritis, Rheumatoid"[Mesh]) AND ("CDP870" [Supplementary Concept])

#1 OR #2 OR #3 OR #4 OR #5

Limits applied: “Adult: 19+ years”,“Humans”,“randomized clinical trial”,“Controlled clinical trial”,“clinical trial”,“clinical trial, phase I”, “clinical trial, phase II”, “clinical trial, phase III”, “clinical trial, phase IV”. There was no limit of language and MeSH terms were searched in “all fields”.

2. In CENTRAL, until 31 December 2010, the search has been conducted using the different names of the drugs and using the term “random*”:

#1 [“infliximab” OR “cA2” OR “TA-650” OR “remicade”] AND “random*”

#2 [“etanercept” OR “TNFR:Fc” OR “(p75)-Fc” OR “enbrel”] AND “ random*”

#3[“adalimumab” OR “D2E7” OR “humira”] AND “random*”

#4 [“certolizumab” OR “CDP870” OR “cimzia”] AND “random*”

#5 [“golimumab” OR “CNTO 148” OR “simponi”] AND “random*”

#1 OR #2 OR #3 OR #4 OR #5

No other limit than the final date was applied.

3. In ISI Web of Science, until 31 December 2010, the search has been conducted using the different names of the drugs, the term “random*” and the indication “rheumatoid arthritis”:

#1 [“infliximab” OR “cA2” OR “TA-650” OR “remicade”] AND “random*” AND “rheumatoid arthritis”

#2 [“etanercept” OR “TNFR:Fc” OR “(p75)-Fc” OR “enbrel”] AND “ random*” AND “rheumatoid arthritis”

#3 [“adalimumab” OR “D2E7” OR “humira”] AND “random*”AND “rheumatoid arthritis”

#4 [“certolizumab” OR “CDP870” OR “cimzia”] AND “random*”AND “rheumatoid arthritis”

#5 [“golimumab” OR “CNTO 148” OR “simponi”] AND “random*”AND “rheumatoid arthritis”

#1 OR #2 OR #3 OR #4 OR #5

No other limit than the final date was applied.

4. Search of unpublished trials: references of meta-analyses in MEDLINE:

("infliximab" [Supplementary Concept]) OR ("TNFR-Fc fusion protein" [Supplementary Concept]) OR ("adalimumab" [Supplementary Concept]) OR ("golimumab" [Supplementary Concept]) OR ("CDP870" [Supplementary Concept])

Limits applied: “Adult: 19+ years”,“Humans”,“Meta-Analysis”.

5. Search of unpublished trials: references of reviews in The Cochrane database of Systematic Reviews:

#1 [“infliximab” OR “cA2” OR “TA-650” OR “remicade”] AND “rheumatoid arthritis”

#2 [“etanercept” OR “TNFR:Fc” OR “(p75)-Fc” OR “enbrel”] AND “rheumatoid arthritis”

#3 [“adalimumab” OR “D2E7” OR “humira”] AND “rheumatoid arthritis”

#4 [“certolizumab” OR “CDP870” OR “cimzia”] AND “rheumatoid arthritis”

#5 [“golimumab” OR “CNTO 148” OR “simponi”] AND “rheumatoid arthritis”

#1 OR #2 OR #3 OR #4 OR #5

6. Search of unpublished trials: meeting abstracts:

For electronic versions (American College of Rheumatology, 2009- 2010 and European League against Rheumatism, 2001-2010) the search was conducted with the different terms: “infliximab”, “cA2”, “etanercept”, “TNFR:Fc”, “(p75)-Fc”, “adalimumab”, “D2E7”, “certolizumab”, “CDP870”, “golimumab”, “CNTO 148”, “remicade”, “humira”, “enbrel”, “cimzia”  and“simponi”.

For print versions(American College of Rheumatology, 1990- 2008), index of each supplement was consulted with the terms: “infliximab”, “cA2”, “etanercept”, “TNFR:Fc”, “(p75)-Fc”, “adalimumab”, “D2E7”, “certolizumab”, “CDP870”, “golimumab”, “CNTO 148”, “remicade”, “humira”, “enbrel”, “cimzia”  and “simponi”, “rheumatoid arthritis, treatment”, “biotherapies”, “immunotherapies”, “TNF-alpha antagonists”, “anti-TNF therapies” and “TNF-alpha”.

7. Search of unpublished trials on clinicaltrials.gov:

#1 [“infliximab” OR “cA2” OR “TA-650” OR “remicade”] AND “rheumatoid arthritis”

#2 [“etanercept” OR “TNFR:Fc” OR “(p75)-Fc” OR “enbrel”] AND “rheumatoid arthritis”

#3 [“adalimumab” OR “D2E7” OR “humira”] AND “rheumatoid arthritis”

#4 [“certolizumab” OR “CDP870” OR “cimzia”] AND “rheumatoid arthritis”

#5 [“golimumab” OR “CNTO 148” OR “simponi”] AND “rheumatoid arthritis”

#1 OR #2 OR #3 OR #4 OR #5

The following limits were applied: “Closed Studies” (in the field“Recruitment”),“All Studies” (in the field “Study Results” (*i.e.* whatever the results of the study, positive or negative), “Interventional Studies” (in the field “Study Type”),“Adult (18-65)”and“Senior (66+)” (in the field “ Age Group”),  “ Phase I”, “Phase II”, “Phase III”and“Phase IV” (in the field “Phase”), 31 December 2010 for the final dates in the field “First Received”and“Last Updated”. There was no limit for patient sex, study sponsor, country and objective.
